# Thrombin generation capacity is enhanced by low antithrombin activity and depends on the activity of the related coagulation factors

**DOI:** 10.1186/s12959-022-00388-w

**Published:** 2022-05-18

**Authors:** Takumi Tsuchida, Mineji Hayakawa, Shota Kawahara, Osamu Kumano

**Affiliations:** 1 Department of Emergency Medicine, Hokkaido University Hospital, N14W5 Kita-ku, Sapporo, 060-8648 Japan; 2 Sysmex Corporation, 4-4-4 Takatsukadai, Nishi-ku, Kobe, 651-2271 Japan

**Keywords:** Antithrombin, Thrombin generation, Disseminated intravascular coagulation, Coagulation factor

## Abstract

**Background:**

Supplementation with antithrombin (AT) concentrates is now common in the treatment of congenital and acquired AT deficiency. However, there is no established consensus on the target and timing of supplementation. We aimed to elucidate the effects of AT deficiency on the balance between coagulation activation and inhibition using a thrombin generation assay as in vitro global assay.

**Methods:**

Samples were prepared by admixing commercially acquired AT-deficient plasma with < 1% AT activity with pooled normal plasma. The AT activity in each sample was adjusted to 100, 90, 70, 50, 40, 30, 10, 5, and < 1%. A thrombin generation assay was performed in each sample. AT concentrate-spiked samples were also prepared by adjusting the AT activities in four types of the concentrates: one recombinant and three plasma-derived AT concentrates. The final targeted AT activities in the samples were adjusted to 100, 50, 30, and 5% by spiking each concentrate into the AT-deficient plasma. We also prepared samples with five levels of prothrombin time (PT) % in coagulation factors with the AT activity fixed at 30% by dilution by mixing AT-deficient plasma and normal plasma with Owren’s veronal buffer to adjust the coagulation factor activities in several proportions. The theoretical target PT% values were 100, 66, 50, 40, and 30%. A thrombin generation assay was performed on all samples.

**Results:**

The ability to generate thrombin depended on the AT activity, and the amount of thrombin generation was increased as AT was decreased. Additionally, the amount of thrombin generation was changed significantly when AT activity was ≤ 50%, indicating that AT suppressed thrombin generation. In particular, thrombin generation was remarkable when AT activity was < 30%, and it can be assumed that the prognosis is poor due to organ failure from thrombotic tendency.

**Conclusions:**

The results presented in this basic research were found to be consistent with the clinical findings to date. The mechanism by which 30–50% of AT activity is set as the clinical boundary was elucidated by the thrombin generation assay.

## Background

Antithrombin (AT) is a 65 kDa glycoprotein produced in the liver and found in the plasma. Its half-life in the blood of a healthy individual is approximately 65 h, and it is present in a ratio of 4:1:5 in the plasma, endothelium, and extravascular spaces, respectively [1]. The physiological action of AT is to inhibit the function of serine proteases, such as activated factors XI, X, IX, and II, by forming a 1:1 complex with them, thereby suppressing the blood coagulation reaction [2]. Thus, AT is an important physiologic anticoagulant.

Because of its role as an anticoagulant, low AT activity, either congenital or acquired, causes thrombosis. Congenital AT deficiency was reported first by Egeberg in 1965 [3]. AT deficiency is now classified into two types: Type I, in which both antigen quantity and activity are low, and Type II, in which the activity is low, but the antigen quantity remains unaffected [4]. Anticoagulant therapy is needed for treating patients with congenital AT deficiency due to frequent venous thrombosis, which requires supplementation with AT concentrate because heparin alone is ineffective [5].

In addition, AT deficiency can also be acquired due to disseminated intravascular coagulation (DIC), severe sepsis, liver cirrhosis, and nephrotic syndrome [6]. Furthermore, strong relationships between the decreased levels of AT activity and prognosis have been frequently reported in patients with several acquired AT deficiency [7–11].

While the normal concentration of AT in human plasma is approximately 2.57 µmol/L, or 0.125 mg/mL to 0.160 mg/mL, which equates to 80–120% of the AT activity [12–14], the appropriate level of AT activity required to control pathological coagulation activation is unclear. In patients with congenital AT deficiency, the high rate of recurrent thrombosis, which is sometimes fatal, requires continued treatment with oral anticoagulants or antiplatelet agents when patients have a history of thrombosis [15]. AT concentrates are effective in both the treatment and prevention of acute venous thrombosis in patients with AT deficiencies. However, there is no established consensus on the target and timing of the supplementation, which is currently administered at the physician's discretion, depending on the individual’s situation [16, 17]. Moreover, AT replacement therapy for acquired AT deficiency in critically ill patients is still controversial, with some reports showing the ineffectiveness [18] and some showing the effectiveness of the procedure [19]. These differences may be due to the patient's backgrounds (e.g., presence of DIC, concomitant use of heparin, underlying diseases, etc.). A study performed to determine the optimal patients who should receive AT supplementation therapy for DIC due to sepsis suggested that AT should be given to those with very low AT activity (< 43%), and it was not effective in patients with AT activity > 43% [20]. However, there was no indication of an obvious threshold for the commencement of AT supplementation for these patients because the balance between bleeding and thrombosis is complicated, and it is difficult to determine the threshold for therapy. Moreover, there have also been few studies that have investigated the in vitro effects of AT supplementation and the relationship between AT and other coagulation factors. This study aimed to elucidate the effects of AT deficiency on the balance between coagulation activation and inhibition using thrombin generation assays as in vitro global assays.

## Methods

### Effects of AT activities on thrombin generation

Samples for our study were prepared by admixing commercially acquired Cryopep Antithrombin Deficient Plasma (CRYOPEP, Montpellier, France) with < 1% AT activities as AT-deficient plasma with CRYOcheck Pooled Normal Plasma (Precision BioLogic Inc., Dartmouth, Canada) as normal plasma. The AT activity in each sample was adjusted to 100, 90, 70, 50, 40, 30, 10, 5, and < 1% by mixing these plasma products. A thrombin generation assay was performed on each sample.

### Effects of supplementation with AT concentrate used in clinical settings on thrombin generation

The AT concentrate-spiked samples were also prepared by adjusting the AT activities in four types of concentrates. One recombinant AT concentrate, ACOALAN 1800 (Kyowa Kirin, Tokyo, Japan) and three plasma-derived AT concentrates, including NONTHRON (Nihon Pharmaceutical Co. Ltd, Tokyo, Japan), Neuart 1500 (Japan Blood Products Organization, Tokyo, Japan), and Anthrobin P1500 (KM Biologics Co. Ltd, Kumamoto, Japan), were used as the AT concentrates. The final targeted AT activities in the samples were adjusted to 100, 50, 30, and 5% by spiking each concentrate into the AT-deficient plasma. A thrombin generation assay was performed on each AT concentrate-spiked sample.

### Effects of coagulation factor activities on thrombin generation

We also prepared samples with five levels of prothrombin time (PT) % in coagulation factors with the AT activity fixed at 30% or 50% by dilution. These samples were prepared by mixing AT-deficient plasma and normal plasma with Owren’s veronal buffer (Sysmex Corporation, Kobe, Japan) to adjust the coagulation factor activities in several proportions. The theoretical target PT% values were 100, 66, 50, 40, and 33%. Samples with 50% AT activity and PT% values 40% or 33% were adjusted by adding AT concentrate, as it was not possible to mix the three samples. Samples with 50% AT activity and PT% values below 40% were prepared by titrating a small amount of plasma-derived AT concentrates (Neuart 1500). A thrombin generation assay was performed on each sample.

### Thrombin generation assay

The thrombin generation assay was described previously in detail [21]. Briefly, thrombin generation was assessed using a calibrated automated thrombogram (Thermo Thrombinoscope, Finggal Link Co. Ltd., Tokyo, Japan). Stimulated thrombin generation was measured using the normal calibrated automated thrombogram method. Plasma samples (80 μL) were supplemented with 20 μL of the PPP-Reagent (Finggal Link Co. Ltd.), containing a mixture of phospholipids and tissue factors. At the start of the measurement, 20 μL FluCa-Kit (Finggal Link Co. Ltd.), containing HEPES (pH 7.35), calcium chloride, and fluorogenic substrate, was added automatically to the plasma samples supplemented with the PPP-Reagent. A FluoroScan Ascent fluorometer (Finggal Link Co. Ltd.) was used to obtain measurements every 10 s; the maximum measurement time was 60 min, and the data were analyzed using Thrombinoscope software (Finggal Link Co. Ltd.). To correct for inner-filter effects and substrate consumption, each measurement was corrected with respect to the fluorescence curve obtained from a mixture of the sample plasma with a fixed amount of the thrombin–α2-macroglobulin complex (Thrombin Calibrator; Finggal Link Co. Ltd.). All the samples and calibrators were run at least in duplicate.

### Data analysis

The peak height and the time to peak calculated from the raw data in the software were used as the parameters for the comparison among samples. In addition, the total amount of thrombin generation during the measurement times was also calculated as cumulative values by integrating the thrombin generation in each detection time. The following parameters were also established to standardize the results and interpret the data for the comparisons.

Relative value (peak) (%) = 100 × the peak height value for each sample / the peak height value of the sample with AT activities < 1%

The relative value (total) (%) = 100 × the total amount of thrombin generated by each sample / the total amount of thrombin generated by the samples with AT activities < 1%

## Results

### The effects of AT activities on thrombin generation

**Fig. 1:**
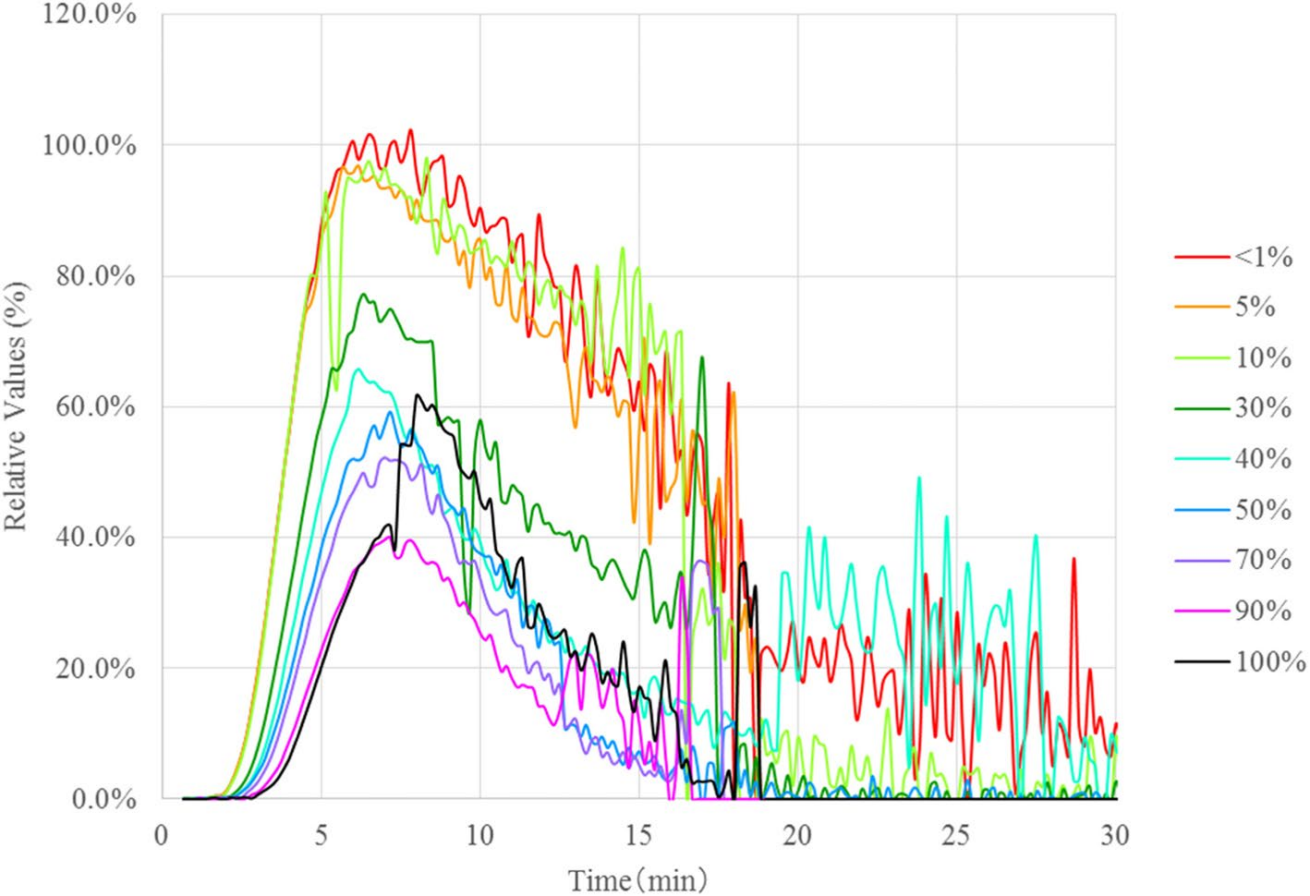
The effects of antithrombin (AT) activity on thrombin generation. For comparison among samples, the peak height value of the sample with AT activity < 1% is defined as 100%, and the following "Relative Value (%)" is calculated. Relative Values (%) = 100 × Parameters for each sample / The peak height value of the sample with AT activity < 1%

**Fig. 2:**
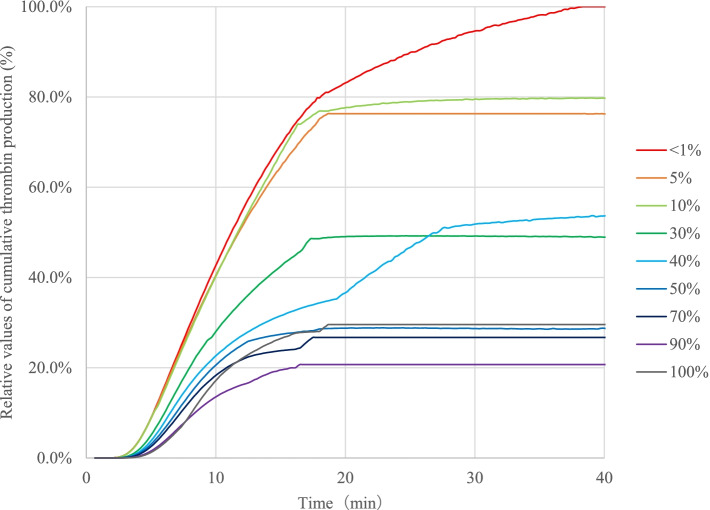
Cumulative thrombin production in antithrombin-deficient plasma mixed with normal plasma. The cumulative thrombin production values for each sample are indicated as relative values with the peak value of the cumulative value for the sample with AT < 1% as 100%. Relative values of cumulative thrombin production (%) = 100 × Cumulative value for each sample / The peak value of the cumulative value for the sample with AT < 1%. AT: antithrombin

The relative value (peak) results of the thrombin generation assays for the samples prepared by the mixing of the AT-deficient plasma and normal plasma in AT 100% to < 1% were compared to determine the relationships between the results of the assays and the AT activities (Fig. 1). Although the results in the samples with AT 100% were higher than the expected values, the graphs of the thrombin generation capacity tests showed a dependence on AT activities, indicating that the thrombin generation capacities were increased when AT activity was decreased. The relative values (peaks) were approximately the same in the range of 10% to < 1%, and there were no significant changes in the graphs; however, the values in the range were increased markedly compared with those in the samples with AT activities of > 30%. The relative value (total) results in these samples are shown in Fig. 2. Similar to the results of the relative values (peaks), no significant changes were observed in the range of < 1–10%, and these values were also significantly higher than those in the samples with the range of 30 − 100%. These results indicated that the generation of thrombin was increased when the AT activities were low and that the increase was remarkably higher in the samples with < 30%.

### The effects of supplementation with AT concentrate used in clinical settings on thrombin generation

**Fig. 3:**
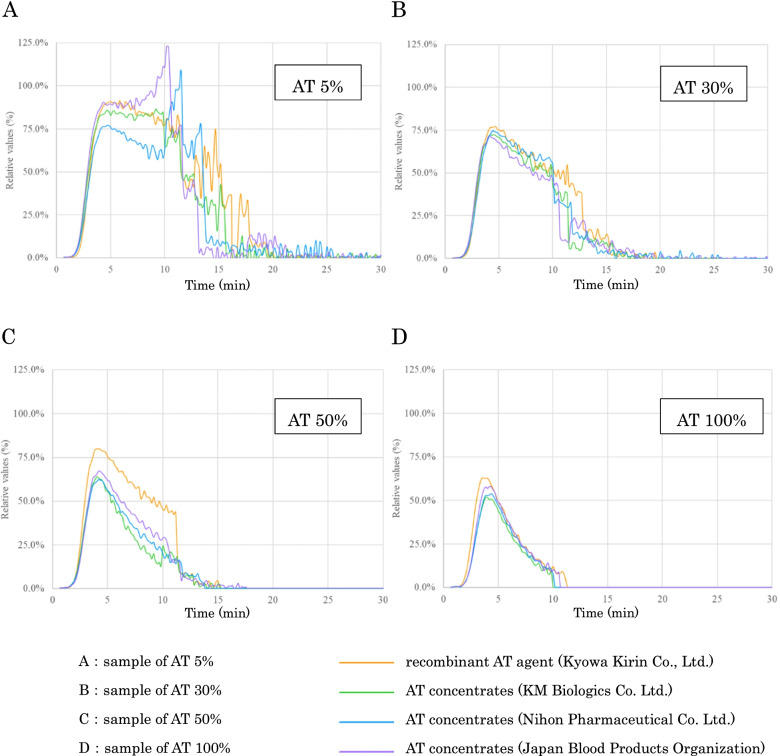
Changes in thrombin generation by the mixture of antithrombin-deficient plasma with antithrombin concentrate. Samples with 5% (**A**), 30% (**B**), 50% (**C**) and 100% (**D**) AT activity are prepared by mixing AT-deficient plasma with AT concentrates. For comparison among samples, the peak height value of the sample with AT activity < 1% is defined as 100%, and the following "relative value (%)" is calculated. Relative Values (%) = 100 × Parameters for each sample / The peak height value of the sample with AT activity < 1%. AT: antithrombin

The relative values (peak) were compared among four types of AT concentrate supplementations in the AT-spiked samples with 5, 30, 50, and 100% AT activities in the same AT-deficient plasma conditions (Fig. 3). These results also indicated that thrombin generation was decreased as the AT activities were increased, and this tendency was similar to those in the results obtained with the samples mixed with normal and AT-deficient plasma. Overall, no significant differences were observed among the four AT concentrates, and the time to peak values were also approximately the same regardless of the AT activities.

**Fig. 4:**
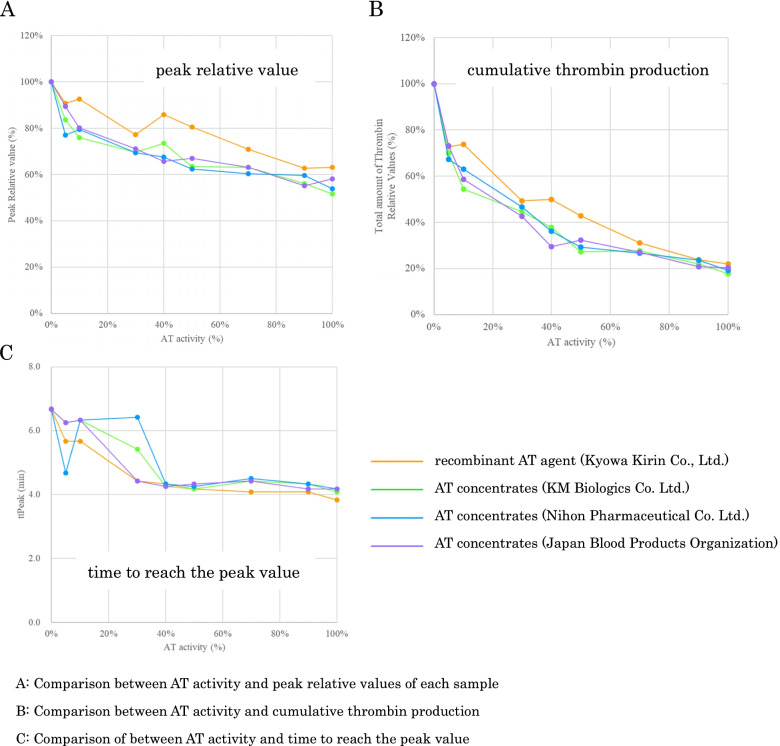
Antithrombin activities, peak relative values, times to reach the peak values, and cumulative thrombin production. The values of the vertical axes are as follows: Peak relative value (%) = 100 × Parameters for each sample / The peak height value of the sample with AT activity < 1%. Total amount of thrombin relative values (%) = 100 × Parameters for each sample / The peak value of the cumulative value for the sample with AT < 1%. ttPeak: time to reach the peak value (min); AT: antithrombin

Figure 4 shows the relationship between the AT activities and the relative values of the peaks, totals, and the times to peak in each graph. It was confirmed that the values of all three parameters were increased as the AT activities were decreased. With regard to the times to reach the peak values, no apparent change was confirmed when the AT activities were ≥ 40%. Although the values of the peaks and total parameters in ACOALAN 1800 were a little higher than those of other concentrates, the tendencies were similar to those in the other three concentrates.

### The effect of coagulation factor activities in thrombin production

**Fig. 5:**
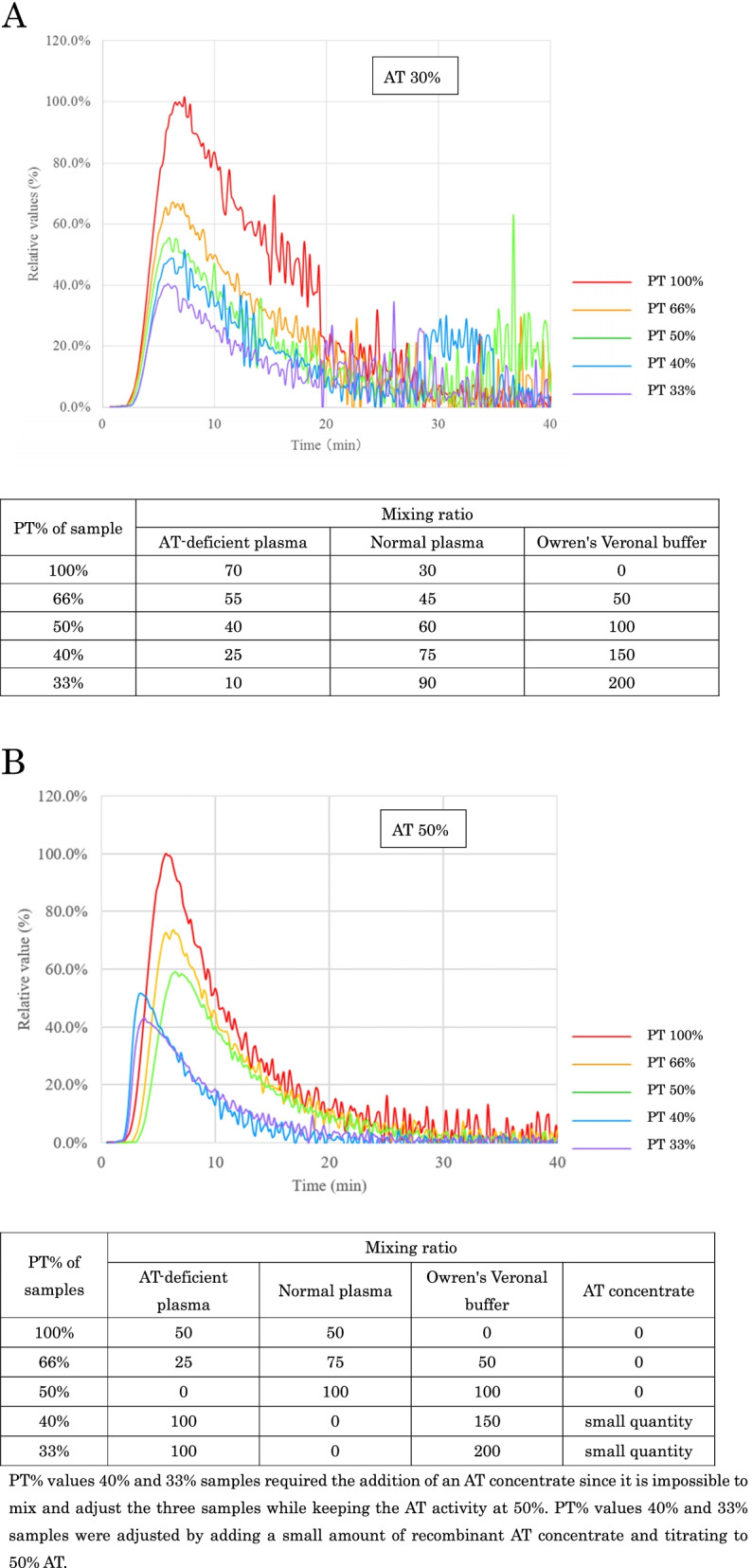
Effect of changes in coagulation factor activity on thrombin generation. This test is conducted at 30% and 50% AT activity. The adjustment method for each sample is as shown in the table above. Relative Values (%) = 100 × Parameters for each sample / The peak height value of the sample with PT 100%. **A**: Effect of changes in coagulation factor activity on thrombin generation at 30% AT activities. **B**: Effect of changes in coagulation factor activity on thrombin generation at 50% AT activities. PT% values 40% and 33% samples required the addition of an AT concentrate since it is not possible to mix and adjust the three samples while keeping the AT activity at 50%. PT% values 40% and 33% samples were adjusted by adding a small amount of recombinant AT concentrate and titrating to 50% AT. AT: antithrombin, PT: prothrombin time.

**Fig. 6:**
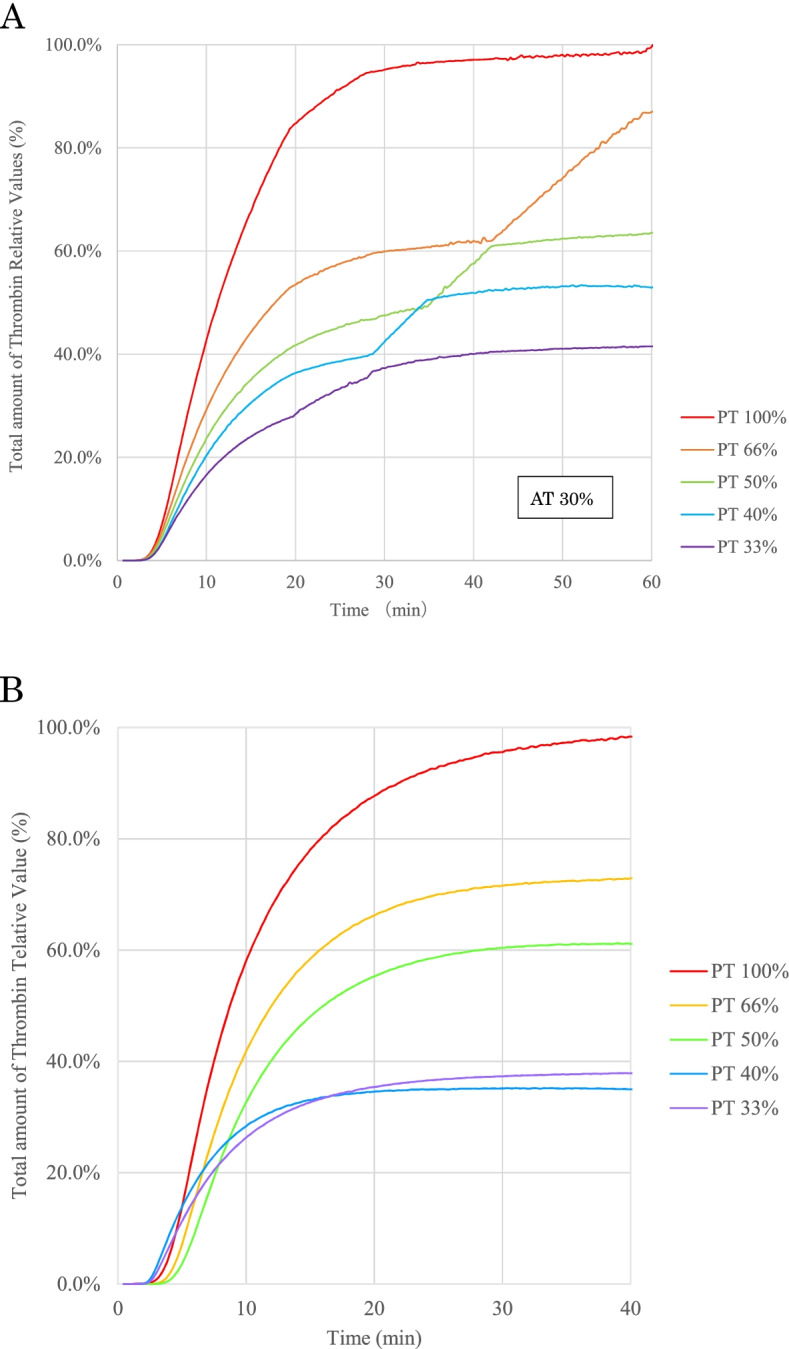
Effect of changes in coagulation factor activity on cumulative thrombin production. This test is conducted at 30% and 50% AT activity. **A**: Effect of changes in coagulation factor activity on cumulative thrombin production at 30% AT activity. **B**: Effect of changes in coagulation factor activity on cumulative thrombin production at 50% AT activity. AT: antithrombin, PT: prothrombin time. Total amount of Thrombin Relative Values (%) = 100 × Cumulative value for each sample / The peak value of the cumulative value for the sample with PT 100%

Since thrombin generation was increased dynamically at < 30% of the AT activities, the effects of related coagulation factor activities were investigated to compare the thrombin generation in the five PT%-level samples at AT 30% (Fig. 5A). With an increase in the coagulation factor activities, a gradual increase in total thrombin generation was observed. The time to peak was constant in each sample. Similar experiments were performed at 50% of the AT activities (Fig. 5B), and the increase in total thrombin generation was observed only when the PT% was above 50% (Fig. 5B). There was more than a two-fold difference in total thrombin generation between 100 and 33% coagulation factor activities (Fig. 6A and B). Therefore, although AT inhibited thrombin generation, its effect was highly dependent on the coagulation factor activities in the plasma.

## Discussion

In this study, the following results were obtained: 1) As AT activities were decreased, the amount of thrombin generation was increased and changed significantly at 10–50% of the AT activities, and it was considered that the threshold was 30%, 2) The type of AT concentrate supplementations did not affect thrombin generation, 3) The time to the peak of thrombin generation was approximately constant regardless of the AT activities, and 4) Total thrombin generation was increased with increasing coagulation factor activities.

The molecular weight of prothrombin is 72 kDa, and its concentration in plasma is 100 µg/mL [22]; thus, its molar concentration is 1.39 mmol/L. Since the molar concentration of AT is 2.57 mmol/L [12], it indicates that the AT molar concentration is approximately two-fold that of prothrombin. The AT forms a complex with thrombin in a 1:1 proportion. When the AT concentration is in excess of that required for thrombin inhibition under physiological conditions, then it is considered that the AT would be able to suppress the thrombin activity quickly due to the higher than sufficient molar concentrations. When the AT activities are decreased to below 50%, the thrombin generation capacity may overcome the suppression activity by the AT because the AT molecule concentrations would be insufficient to inhibit the generated thrombin. In the present results, a significant increase in thrombin generation was observed below 50% of the AT activities, which was consistent with the theoretical values calculated from the molar concentrations. In particular, thrombin generation was increased remarkably when the AT activities were < 30% (Fig. 1), and it was considered that the threshold line of low AT activities was 30%.

An AT activity threshold line was also investigated in several studies, and the decreased levels of AT activities have been shown to be associated significantly with an increased mortality in patients with sepsis, with < 50% of AT activities showing a sharp increase in mortality due to organ failures from thrombotic tendencies [23]. In terms of finding a target area during clinical AT replacement therapy, it has been reported that it was effective in septic DIC in cases where the AT activities were < 43%, instead of 70% [20]. The threshold line established in the clinical studies was similar to the one in our study; it was considered that our study performed in vitro reflected the physiological conditions and the thrombotic tendencies caused by high thrombin generation derived at low AT activities. Moreover, monitoring of the AT activities during AT supplementation has been reported to be useful in predicting patient outcomes [24]. In our study, the results showed that low AT activities resulted in increased thrombin generation, and it was considered that these data reflected the thrombin generation reaction in vivo. Therefore, a thrombin generation assay may find the potential thrombosis risk in low AT activity patients and be useful in predicting the prognosis of patients receiving AT supplementation. Recently, during the coronavirus disease 2019 (COVID-19) pandemic, many studies revealed that the thrombosis tendency was often seen in COVID-19 patients and that the incidence was higher in severe cases [25]. These severely ill patients presented with coagulopathies, and Tang et al. reported that 71.4% of the non-surviving COVID-19 patients fulfilled the criteria of DIC, whereas only 0.6% of the survivors met the criteria [26]. It was also reported that since the AT activities were maintained to approximately 80%, supplementation of the AT was not necessary in most of the cases [27]. However, a low AT activity is one of the risks of thrombosis. To prevent coagulopathy in COVID-19 patients, AT activity measurement is necessary, and a thrombin generation assay might be useful in predicting thrombosis tendencies.

Under conditions in which AT was fixed at 30% (Figs. 5A and 6A), thrombin generation was increased proportionally, with an increase in the concentration of coagulation factors, especially for prothrombin. This is because thrombin production increased in proportion to each prothrombin concentration, since the conditions in which PT was present were more than that of AT (PT% ranged from 33 to 100%). In contrast, under conditions in which AT was fixed at 50% (Figs. 5B and 6B), thrombin production increased only when PT% exceeded 50%. At 33% and 40% PT, thrombin production was low because AT activity sufficiently exceeded prothrombin concentration; when PT% exceeded 50%, thrombin production increased with prothrombin concentration (PT%) because of the dominance of prothrombin. It can be suggested that using global assays, the thrombin generation reflected the balance between the amount of coagulation factors, including prothrombin and AT activities. Although the thrombin generation and cumulative production increased in normal PT%, the values of time to peak were at the same levels in five different PT% activity samples. It means that the time from coagulation cascade activation to thrombin generation was not so different in these samples, and the time to peak values were independent of the activity of coagulation factors. Theoretically, AT inhibits not only thrombin but also other serine proteases, such as activated factors XI, X, and IX. The similar values of the times to the peaks indicated that the inhibition proportion of AT to these serine proteases did not change in the five different PT% activity samples because the times from activation to thrombin generation could be prolonged if AT inhibited these serine proteases in the earlier phase of coagulation cascade. The thrombin generation assay system may evaluate the effects of coagulation factors in addition to AT.

Conventionally, plasma-derived AT concentrates derived from human plasma have been used for the AT concentrates; however, in recent years, recombinant AT concentrates, which suppress the risk of transmission of infectious diseases, have been employed. It has been previously reported that there were no differences shown in the administration of recombinant AT concentrates or plasma-derived AT concentrates in healthy volunteers or DIC patients in clinical studies [28, 29], and the present results also showed no differences between the different types of AT concentrates. Thus, it was considered that the function of the recombinant AT concentrates was the same as that of the plasma-derived concentrates, and the same threshold line could be used for predicting patient outcomes and the monitoring of AT activities.

### Limitations

The number of samples used in this study was small. Furthermore, the results may be biased due to the use of only specific plasma samples. We also showed that the threshold established from the thrombin generation assay was similar to that shown in clinical studies. Although it was considered that the assay reflected the physiological conditions, there were many cases with low AT activities. In this study, the in vitro and in vivo data were insufficient to draw comparisons.

## Conclusions

The findings of this study were consistent with the clinical findings of other studies that have been conducted to date. The decision to use 30–50% of the AT activities as the clinical boundary was determined using the thrombin generation assay. In the future, it is expected that appropriate AT replacement therapy will be established by further elucidating the pathogenesis and that the point-of-care testing of coagulo-fibrinolysis will be established by the development and widespread use of thrombin generation assays.

## Data Availability

The corresponding author can disclose the data when requested.

## Abbreviations

AT: Antithrombin
DIC: Disseminated intravascular coagulation
PT%: Prothrombin time % activity
